# Tribological Behavior of Friction Materials of a Disk-Brake Pad Braking System Affected by Structural Changes—A Review

**DOI:** 10.3390/ma15144745

**Published:** 2022-07-06

**Authors:** Filip Ilie, Andreea-Catalina Cristescu

**Affiliations:** 1 Department of Machine Elements and Tribology, University Politehnica of Bucharest, 060042 Bucharest, Romania; 2 Department of Biotechnical Systems Engineering, University Politehnica of Bucharest, 060042 Bucharest, Romania; andreea.catalina.cristescu@gmail.com

**Keywords:** brake disk-pad, braking materials, structural changes, tribological performances

## Abstract

For road safety, braking system performance has become a very important requirement for car vehicle manufacturers and passengers. To this end, vehicle designers must understand the characteristics of tribological behavior and the causes of their variation in properties. This paper analyzes the tribological behavior (at friction and wear) of the most recent material couples of the braking disk-pad system affected by their structural change through the implications on the braking system stability, reliability and suitable characterizations. Obtaining information to design a very efficient braking system and assessing the influence of the material’s structural changes on its stability has become a necessity. This has been made possible by using several methods of testing a brake disk-pad couple on various devices intended for this purpose. The materials of the contact surface disk-brake pad with their tribological performance (friction, wear), especially the friction coefficient, present particular importance. Also, system components’ reliability, heat transfer and the noise and vibration of the brake disk-pad couple are vital to the correct operation of the braking system and should be given special attention. The test results obtained define the friction patterns and the influence of structural changes and other environmental factors that can be used in computer analysis.

## 1. Introduction

The advancement of technology in order to achieve braking systems, with improved performance continues, in order to ensure safe and stable braking. Such improvement has been brought to the disk braking system by the addition of disks of ferrite magnets, reducing both the time and the braking distance, resulting in safe and smooth braking even at high velocities [1].

Several studies and researches have been performed for the purpose of improving the braking system performance of motor vehicles [2,3] through:

- materials used, in particular, for those of contact surfaces;
- design of the whole assembly of the braking system including the component parts;
- braking system behavior over time, assessed using simulations and field testing;
- drive type of the braking system: mechanical, electric, electro-mechanical regenerative, magnetic, hydraulic, pneumatic or vacuum.

A braking system is essential for the proper operation in road traffic conditions and the safety of a vehicle. Over time, the reliability and efficiency of braking systems have been continuously improved [4]. The braking couple represents the main feature of these systems, and its reliability and durability, or other braking system components, ensure a reliable braking function, without interruptions or defects [3].

Velocity control is the brake’s main function, and the energy dissipation rate defines the deceleration rate of the vehicle. The braking system elements must increase the force exerted on the brake pads by the driver’s foot. As a result, supplementary control and assistance systems were added to the basic equipment of the braking system, with the purpose of helping the driver in difficult and critical situations and to provide increased comfort in traffic conditions [5].

**Figure 1:**
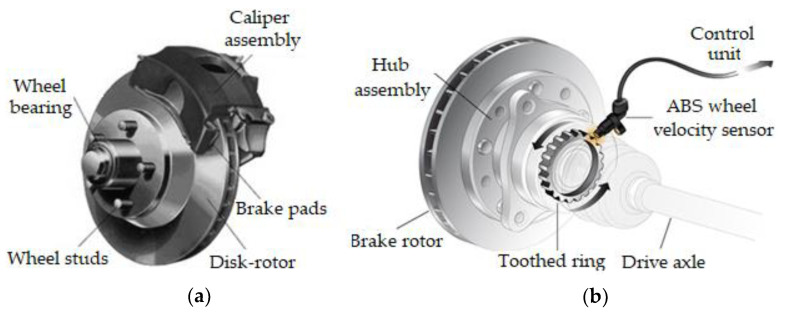
Braking systems; (**a**) pads with disk; (**b**) Antilock Braking System (ABS).

Lately, to respond as well as possible to traffic conditions, especially those related to heat transfer at brakes [6], cars have been equipped with an Antilock Braking System (ABS), in addition to braking systems with disk-brake pads (Figure 1a,b).

Hence, the braking system of a modern machine consists mainly of disk-brake pads on the front and rear axles, together with a connection system that engages the main and secondary cylinders with the calipers [7]. Various issues, such as the system components’ reliability, noise and vibration, the disk-pad braking materials couple, the heat transfer and heat flux must be known for the proper operation of the braking system. Knowledge of the temperature and heat flux at the interface between braking materials couple and at their boundary with the environment is of critical importance to safety engineering (here road safety), mechanical engineering and in many other engineering fields [6,7,8].

Talati and Jalalifar [9] studied heat transfer in the braking system and identified the governing equations in time and space for the transition heat transfer of the brake disk and pad. Nosko et al. [8], to determine the temperature at a sliding surface, developed an inverse heat conduction algorithm in the case of friction of brake material on steel. Ganji and Ganji [10] analyzed the brake squeaking phenomenon from the point of view of amplitude and frequency of the oscillations in a limited cycle, considering nonlinear equations of motion due to large distortions [9] using the Stribeck friction model. The parameters that affect brake noise are the subject of ref. [11], where Phatak and Kulkarni achieved squeaking reduction by the stiffening and structural modification of the brake pads. A study of the performance parameters and the functional characteristics of braking system components was presented by Ebrahimi-Nejad and Kheybari in ref. [12]. They carried out a detailed study based on the data and specifications in ref. [12] of a specific braking system, designed according to the regulations and standards in ref. [13]. Applying the digital logic method to the optimized material selection procedure has been studied and revised in various material selection schemes [12]. Wei et al. [14] made stratified brake pads from two materials: two layers of plastic (the first and third) and one layer (the second) of felt.

Kumar and Kumaran [15] and Perez and Echeberria [16] have proposed a preparation process for the friction material of the brake pad of sintering a metal powder blend which is then cold-pressed. This process improves adhesion and tribological properties (friction coefficient and wear resistance). Also, Wei et al. [14] have proposed a preparation process for the friction material of the brake pad of sintering a metal powder blend which is then cold-pressed This process improves the adhesion and the tribological properties (friction coefficient and wear resistance) as well as stiffness, damping properties and braking system stability. However, the dynamics of the braking system remain unclear [14].

On the other hand, Wei et al. [14] moved from a three-layer brake pad to a two-layer one which consisted of a base layer as support and a friction layer that comes into contact with the disk where the braking force appears. The stability analysis, characteristics of the two layers (friction and support) of the brake pads and the pad parameters optimization further improved braking system stability and reduced the occurrence of chaotic vibration [14].

Due to increasingly demanding road safety requirements, braking system performance has become a very important condition for both car manufacturers and passengers. Therefore, it has become a necessity to assess the structural changes that influence braking system stability and the information required to choose the best braking system design.

The purpose of this paper is to analyze the latest material couples of the braking disk-pad system, their structural changes in correlation with the main tribological aspects of friction and wear and the implications for braking system stability and reliability and friction coefficient evolution.

## 2. Disk and Pad Brake System Friction Materials Specifications

Friction materials for car brakes, especially for the disk and pad brakes, are defined according to the material used, namely: non-asbestos organic materials, ceramic materials, semi-metallic, hybrid, low metal content, etc. Some of these trade names, such as the organic non-asbestos materials formulas are very important, but there is no conceptual difference between several formula types, in most cases, beyond the names.

The gradual elimination of asbestos was acheived in successive stages. The first challenge with which materials engineers were confronted was finding those fibers which could successfully replace asbestos which, due to its multitude of favorable properties, had become the most widespread and popular material among friction materials. At first, it was difficult to find a replacement for this material because the new materials did not fully match its properties. This was the case with organic fibers and steel fibres, but altering their properties through the use of many raw materials allowed the development of complex formulas for asbestos replacement [13].

It is known that the friction material represents only one element of the braking system. Essentially, vehicle brakes are composed of disks or drums made from cast iron or steel, and most friction materials were developed and designed to use cast iron as the contact surface. Hence, the precise metallurgical composition of cast iron and its type, play a very important role in the braking system [17,18].

**Figure 2:**
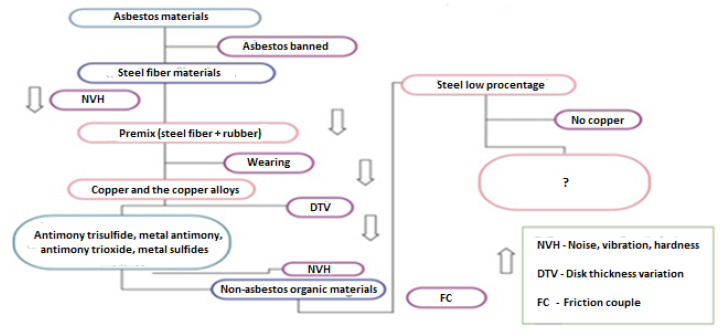
Presentation of the friction material formulas evolution from the last two decades (reprinted/adapted with permission from ref. [21]. 2022, Elsevier).

Copper (in the form of fiber and powder), as well as steel fibers, have become two important friction materials. Often copper’s importance near-surface is unclear, but its role is related to high thermal conductivity. Copper transfer on the composite material disk as a tribofilm is a well-known phenomenon [19]. The evolution of friction materials is shown in Figure 2.

There is a lot of research and studies into friction materials with different sulfides and metals used today, including into the decreased percentage of heavy metals used in the material composition [19]. The choice of friction material is of paramount importance, as shown in Table 1. Disk brakes in cars are designed mainly in cast iron, and for motorcycles are made of stainless steel. This presents a major problem because their performance in humid conditions is substantially lower than in dry conditions, by a factor of up to three times (i.e., from a friction coefficient of 0.6 to 0.2, see Table 1).

This difference in braking performance is not acceptable and therefore, on disks and pads, the manufacturers make grooves and/or slots to help remove the water film from the friction surface. For operation in conjunction with stainless steel disks, friction materials with special formulations were developed [19]. When the metal percentage exceeds (typically) 50% of the total mass of the friction material they are defined by the so-called semi-metallic formulas. Metal components consist of copper or its alloys (bronze and brass), steel fibers, and in some cases tin and zinc, while others are a mixture of lubricants and strong abrasives [20]. Raw components of asbestos-free friction materials with the percentage content of lubricants and metals are shown in Table 2.

**Figure 3:**
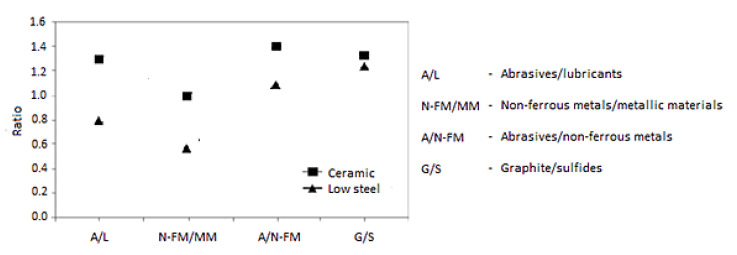
The classical ratio between the main friction materials categories.

Figure 3 presents some classical ratios between the main components of the friction materials: metallic materials (including steel fibers), abrasives, lubricants, sulfides and graphite for the ceramic formulas, non-ferrous metals, semi-metallic and low alloy steel. The abrasives in the composition of the ceramic materials are only weak abrasives (zirconium silicate), while in the composition of the semi-metallic materials, abrasives are usually strong (alumina and less often corundum), because upon disk contact surfaces oxides are formed, which must be removed [21,22].

**Figure 4:**
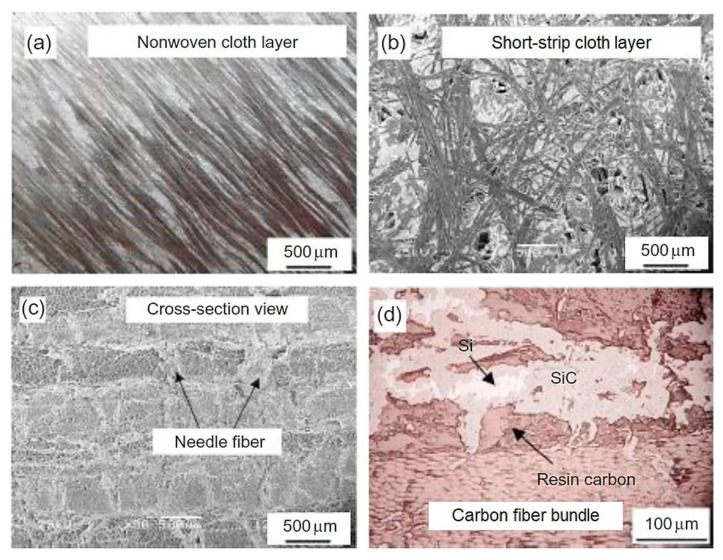
Images of C/C-SiC composite microstructure: (**a**) nonwoven cloth layer, (**b**) short-strip cloth layer, (**c**) cross-sectional view with needle fiber, (**d**) the different components of the final composite material (reprinted/adapted with permission from ref. [21]. 2022, Elsevier).

In the last decade, carbon-ceramic materials, mainly carbon/carbon-silicon carbide (C/C-SiC), were used as friction materials and their microstructure is shown in Figure 4 in various stages of manufacture and with a density of 2000 kg/m^3^. Carbon-ceramic brake disks are frequently used for state-of-the-art machines, although they are much more expensive than other materials, but more accessible than carbon-carbon materials [21,22].

C/C-SiC composite mechanical properties are shown in Table 3. Of note is that the perpendicular direction strength on the sliding surface is higher than those in the parallel direction (see Table 3), due to the participation in the mechanical strength of both matrix and fiber frameworks [23]. For compression strength in the perpendicular direction, the load is supported both by matrix and fibers. The composite material, in this case, has an elastic behavior until material failure (being tenacious).

C/C–SiC materials are suitable for use in braking systems because they exhibit adequate mechanical properties. Due to the existence of significant differences in the parallel and perpendicular directions on the sliding surfaces the mechanical characteristics of these materials are anisotropic. In bending tests, cracks always begin in the matrix and propagate in the fiber direction before, finally, fiber bundles are pulled out. After matrix failure, the exposed fibers, which bear all the load, yield the consequent destruction of the composite. The material exhibits a pseudo-plastic behavior in bending tests, without a sharp load fall.

There are significant differences between the different types of C/C–SiC products caused by the production process and depending on the final porosity and composition.

## 3. Materials and Methods

The friction materials used in the tests presented in this paper were composite/cast iron friction couples bound with a resin binder, composite material brake pads and, cast iron disks.

Brake pads made from composites (a mixture of metal powders, graphite and inorganic materials bound with a special resin) are the most popular and suitable for use in every day cars. Such a structure ensures heat transfer (works better in a hot climate, sensitive to low ambient temperatures), high reliability, efficiency, little noise, does not wear the brake disks too much and has a good price/quality ratio. The data provided by friction material manufacturers (operating recommendations, friction and wear characteristics, mechanical properties, and an indication of the peak working temperatures) through the technical specifications, constitute the starting point for specialists in the brakes or vehicle field.

Using a dynamometer tests were carried out to estimate the stability/fade performance, friction levels, noise, and wear performance of the braking system. Usually, frictional performance is estimated through the results of an industry-standard test regarding the nominal value, *μ*, or using a design for which there is associated data regarding variation with temperature. The temperature was measured with a standard sensor placement (a rubbing thermocouple on the disk).

When a brake operates at high temperatures for an extended period, the brake material may be damaged. To avoid this, specialists in vehicles will size the brakes using power density as a design parameter which is a much more useful parameter. Because the type of friction material influences the variation of the friction coefficient with temperature and manufacturers use their own specific measurement methods of temperature, there are contradictory results. Thus, a common and relatively recent method of evaluating variations in surface temperature is using an infrared pyrometer (infrared pyrometry).

Furthermore, to determine the temperature using inverse heat conduction at a sliding surface, a pin-on-disc tribometer was used in the case of friction of brake material on steel. The tests realized on the pin-on-disk tribometer were in a short-time sliding regime (for speed step, acceleration and deceleration). From a brake pad (of a composite material) of a car, was made a pin sample with a 10 mm diameter, and the disk was made from steel. With the help of two thermocouples the temperature of the pin sample was measured. The thermocouples were identical, miniature and installed at different distances from the friction surface.

The measurement of friction coefficient was made on a device for testing brake material in small samples by more tests at a cyclic application interval of a constant normal load for 20 s and a constant sliding velocity of 7.15 m/s (maximum velocity developed by the test device). This method of measurement friction coefficient at different ‘start’ temperatures, measured against a cast-iron disk, highlights composite friction material performance resin-bonded.

For the tested friction materials pair (composite/cast-iron) prolonged slipping at temperatures above 300 °C will lead to changes in the friction material’s surface, friction, wear characteristics and mechanical properties (depending on the operating conditions and material). In other words, friction performance is significantly affected.

## 4. Results and Discussion

In general, Amontons–Coulomb friction laws are valid for friction materials in the braking system, but in the case of a composite/cast iron friction couple bound with a resin binder, the friction coefficient does not remain constant. The physical reasons why the variation of the friction coefficient occurs are useful to understand. The main cause is temperature, because during the brake’s work, it will heat up and increase the friction material temperature. Friction at the interface, following low thermal diffusivity of friction material, generates very high temperatures even in relatively slow operating conditions.

**Figure 5:**
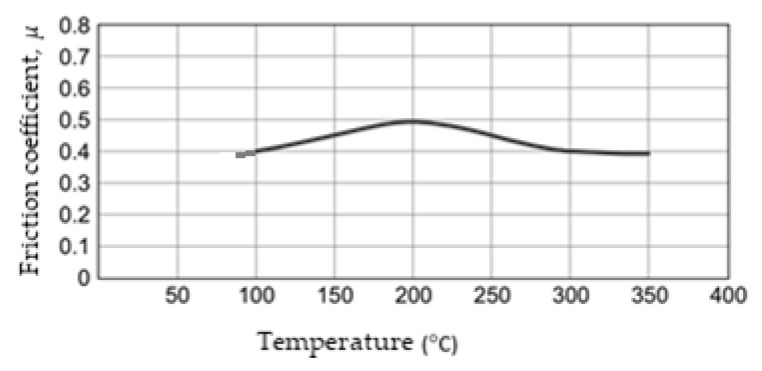
Variation of the friction coefficient of the composite/cast iron couple bound with a resin binder (reprinted/adapted with permission from ref. [13]. 2022, Elsevier).

The resin binder’s thermo-physical properties are heat-strength, but many other constituents will be altered by temperature. The effect is the appearance of chemical reactions and friction material thermal degradation at the interface, and the result is the change of the friction coefficient with temperature. Normally, as shown in Figure 5 (for the composite/cast iron couple bound with a resin binder), the friction coefficient increases slightly with a brake disk temperature of about 200–250 °C and then decreases.

A composite/cast iron couple was used for testing because the brake pads in composites are the most popular. They have a semi-metallic friction layer (composed of one-third to two-thirds metal part; the rest is a mixture of graphite and inorganic materials) and are ideal for equipping cars that run every day.

Composite brake pads are made of a combination of copper, steel, graphite and brass, all bound with a special resin. This type of brake pad offers high reliability and good heat transfer (they have the ability to absorb heat efficiently), they are also very efficient, make little noise and have an optimal quality/price ratio.

On the other hand, they are quite sensitive to low ambient temperatures (so they work better in a hot climate), do not wear the brake disks too much, and when they do wear out, they squeak and brake disk wear begins, or if their hardness is excessive this can lead to premature wear of the brake disk.

Usually, for their friction materials, their manufacturers provide technical specification sheets which present the mechanical properties, friction and wear characteristics and an indication of temperature effects (as peak working temperatures) and the recommended operating conditions.

For brake or vehicle specialists, this data constitutes only a starting point. Tests with a dynamometer are used to evaluate stability/fade performance, noise performance, friction levels and wear performance (between the brake pad and disk).

Based on results from an industrial-standard test, the frictional performance is evaluated, usually in the form of a nominal or design value, *μ*, with a variation associated indication with temperature. The temperature is measured using a rubbing thermocouple on the disk (in the case presented in Figure 5) or an embedded thermocouple in the friction material of the disk using a standard sensor arrangement.

The mechanical properties listed above are usually values based on results obtained from industry-standard tests, where the conditions are specified (e.g., a maximum tensile strength of 15 MPa, maximum shear strength of 25 MPa, Rockwell hardness HRC80 and density of 1950 kg/m^3^, are typical average values). For ‘recommended working peak temperatures,’ such as continous at 250 °C and intermittently 350 °C, there is no industry-standard test and this information is usually empirically obtained with an emphasis on life at wear. The working peak temperature is often exceeded when the operating temperature of a brake on a specific vehicle under any defined brake load, varies with time.

It is noteworthy that the time period spent at high temperatures is very important because this can cause friction material permanent damage. Therefore, a much more useful design parameter used by vehicle designers is power density to size of brakes.

It should be noted that the function of the friction material depends on the precise variation of the friction coefficient with temperature [13,22]. Thus, manufacturers of friction materials prefer their own temperature measurement methods, that not are comparable with the methods used by other manufacturers, obtaining contradictory results. Recently, a good way to identify variations in surface temperature is infrared pyrometry, provided that the surface emissivity issue is overcome.

More recently, Nosko et al. [8], to determine the temperature at a sliding surface, have developed an inverse heat conduction algorithm taking account of thermocouple thermal inertia [24]. This algorithm was applied in the case of brake material friction on steel. By using the Laplace integral transformation the direct heat conduction problem was solved analytically. On a pin-on-disk tribometer, experimental tests were conducted for short-time sliding regimes (regarding speed step, acceleration and deceleration), with a pin sample taken from a brake pad (composite material) of a car, with a steel disk. The temperature in the pin sample was measured with the help of two identical thermocouples fixed at the friction surface but at different distances. It was found that the two inverse temperatures of friction surface are in good agreement with each other. The inverse algorithm estimates contact temperature, which can be measured using infrared thermography, with an accuracy of 5–7%.

As explained above, the temperature increases as the brake is applied, and the friction coefficient changes. To maintain the same test conditions as the reference temperature a ‘start-of-stop’ temperature is taken. On the other hand, the disk temperature is taken as the defining parameter on the initial application of the brake, for comparing different applications of the brake.

**Figure 6:**
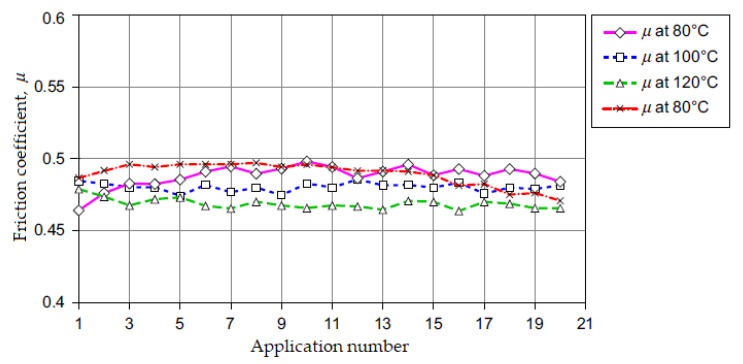
Measurement of the friction coefficient, *μ* in a device for testing brake material samples at different disk temperatures as a function of time and linear slip velocity of 7.15 m/s (reprinted/adapted with permission from ref. [13]. 2022, Elsevier).

More tests for measuring the friction coefficient of the resin-bonded composite friction material on a cast-iron disk (composite/cast-iron couple) were made using a device for testing brake materials in small samples, at an application interval of 20 s, and a constant sliding velocity and are shown in Figure 6. These tests also represent a typical example of performance of the same friction material couple of composite/cast-iron at different ‘start’ temperatures, measured against the cast-iron disk.

These data from Figure 6 show the friction coefficient variation between test sequences and during a sequence. For these tests, we used composite friction material specimens with 10 mm diameter sliding on a cast-iron disk, which rotated with a constant speed equivalent to 7.15 m/s. Then, a constant normal load cyclic was applied(by removal and repetition) for 20 s and for 20 applications (see Figure 6). The first application was made when the disk had reached the required initial temperature of 80, 100 or 120 °C. Furthermore, natural convection cooling was insured.

The first test (at a brake disk start temperature of 80 °C) indicated an increase in the friction coefficient from *μ* ~ 0.46 to 0.49. A brake disk start temperature of 100 °C (the second test) showed a fairly stable friction coefficient, *μ* ~ 0.48. A brake disk start temperature of 120 °C (the third test) showed a friction coefficient that was quite stable and reduced to *μ* ~ 0.46. Finally, the fourth test (returning to a brake disk start temperature of 80 °C) showed an increase in the friction coefficient from *μ* ~ 0.46 (specific to the start temperature of the third test at 120°C) up to the value indicated by the first test at 80 °C (*μ* ~ 0.49), then surprisingly, it returned to the value (*μ* ~ 0.46) of the test at 120 °C [13,22].

**Figure 7:**
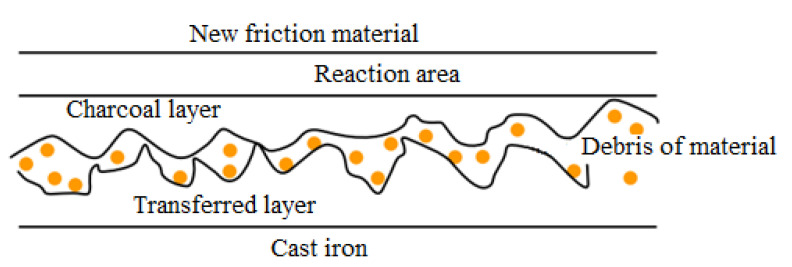
Model of a braking friction couple “five-phase” (cast iron disk and composite friction material), which includes a bond with resin (reprinted/adapted with permission from ref. [13]. 2022, Elsevier).

The friction coefficient reducing with temperature is usually defined as “fading”. The physical explanation of fade is due to creating, essentially, pseudo-hydrodynamic sliding conditions as a result of the resin’s volatile organic components, as well as other constituents, which determines the appearance at the interface of pressurized steam or gas regions, separating the sliding surfaces. The friction performance of new or “green” materials is likely different from that of the friction materials used to date because volatile components are much more abundant in partially hardened friction materials, often with several variations in temperature. As a result, the new friction materials of the braking system must be treated with care and not subject to operation at high temperatures until they have had a chance to accommodate (i.e., to touch the geometric conformity at the friction interfaces polishing, to obtain a constant sliding state or the friction interfaces tribological contact. This implies exposure to the temperature of a new friction material layer to remove volatiles from the reaction area and fully adapt (Figure 7) [13,22].

When a brake friction material is subject to operation at a temperature high enough to cause fading, it is expected that, when allowed to cool, the friction coefficient, *μ* will return to the initial value (see Figure 6). However, there an effect that frequently occurs, known as “delayed discoloration”, where the temperature effect is largely reversible. In extreme conditions, vehicle brakes may be allowed to cool but generate a low friction coefficient value, *μ*, when applied. For a friction materials couple (a typical cast-iron disk associated with resin-bonded composite materials) prolonged slipping at temperatures of above 300 °C (depending on the operating conditions and material) will have an effect on the change in the friction material’s surface, and possibly in the disk thickness of the friction material.

At the same time, the organic components in the friction material’s composition, which control friction and wear characteristics, begin to thermally degrade, the friction performance is significantly affected and the mechanical strength decreases.

**Figure 8:**
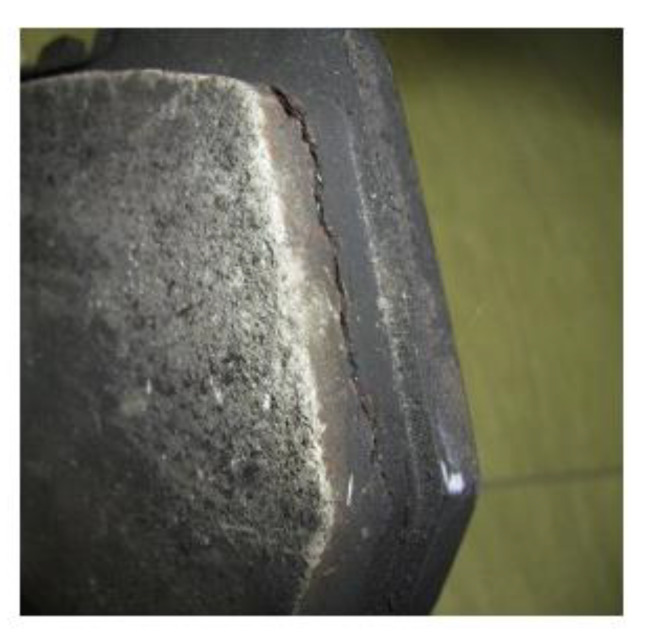
An example of a “distorted” disk brake pad as a result of high temperature and excessive load (reprinted/adapted with permission from ref. [13]. 2022, Elsevier).

Finally, the friction material surface becomes “distorted” because only the temperature-resistant components remain and all the organic constituents are burned (see Figure 8), andfrictional and wear resistance deteriorates irreversibly [23].

Friction performance may also be affected by velocity. It is defined as the existence of a transition area between the static friction coefficient, *μ_s_* and the sliding one, *μ_a_*. Usually *μ_s_* > *μ_a_*, thus, the brakes can operate less at very low velocities, producing creak and vibration effects such as “creep-gem”. The velocity effects are related to thermal conditions and temperature distribution, almost entirely in the case of resin-bonded composite materials. A higher sliding velocity at the friction interface means a higher energy dissipation rate and a higher vehicle velocity. Thus, higher interface temperatures are generated and the friction coefficient, *μ* decreases accordingly. This phenomenon is particularly noticeable in heavy commercial vehicles and is known as “velocity sensitivity”.

**Figure 9:**
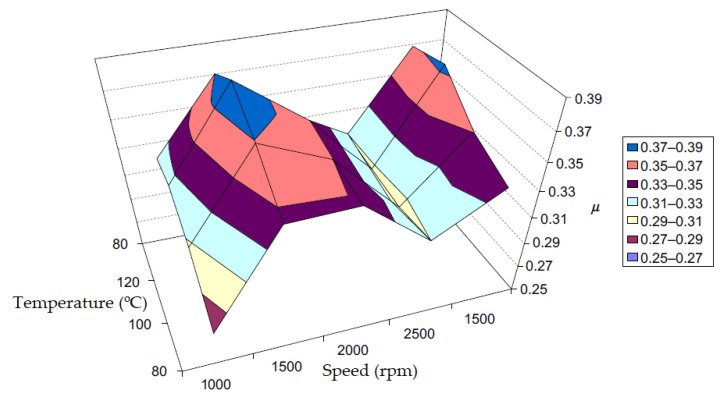
Effect of temperature and velocity on the surface of the friction composite (reprinted/adapted with permission from ref. [13]. 2022, Elsevier).

For a typical composite material bonded with resin and against a cast-iron disk which has been tested on the same small samples device as above, the effects of velocity and temperature are shown in Figure 9. To indicate that friction performance repeatability is mentioned, the velocity axis extends from 1000 to 2500 rpm, then it returned to 1500 rpm. This also applies to the temperature axis extending from 80 to 120 °C, then returning to 80 °C. Repeating an experiment, under the starting conditions, is standard practice in completing a friction material test sequence and in verifying so-called “recovery”. The test results can be used to define friction patterns and for computational analysis [23].

Friction performance can be affected by the existence of many other environmental and operational conditions.

Thus, the vehicle’s brakes can be noisy when humidity is high, such as on cold, damp mornings, and *μ* may rise, but after a few applications the temperature can increase, the water can dry (evaporate) and *μ* returns to a normal operating level. Also, frictional performance may reduce by immersion or soaking in water, due to the presence between the friction surfaces of a lubricating film (liquid or vapor).

The controlled introduction of water on a frictional surface which is very strongly heated can improve brake performance, because the heat dissipation increases through the latent heat of evaporation of the water. How the *μ* variation presented above is related to brake use in the operation regime at the high loads. At the same time, *μ* may also be affected by the brake operation regime with low duty, resulting in low temperatures, and can be associated with low frictional performance (low *μ*). Therefore, surface films should be removed or replaced before steady-state frictional performance may be achieved.

The tribological conditions of the interface when a composite brake pad made of conventional resin is applied and placed on a cast-iron pair surface are different from the ‘steady-state’ conditions existing between worn friction couples. It is specified that the establishment of tribological conditions operating in the steady-state is referred to as “bedding”, or often “polishing”. Thus, when preparing for operation, a new friction couple, two aspects can explain this in more detail, namely [2,22,23]:

(1) In the friction and wear process, geometric conformity will be generated between the two surfaces so that the whole apparent area of the brake pad and disk is in complete contact, which is considered “bedding”. If the brake is in heavy use before the bedding is complete, it is possible thermal deformations may occur and the friction is done on a smaller contact surface, so the friction couple durability decreases (becomes less resistant to wear).
(2) The sliding process between the disk and the friction material will be determined as the friction surfaces are transformed by mechanical, chemical and thermal processes that take place in the friction process until the establishment at the interface of a quasi-stationary tribological contact. The brake pad and disk surfaces will generate transfer films at the interface, which can be friction modifiers, filler (brake powder), polymeric films resulting from binder resin and its components, other substances that appear in the friction process, etc., or the “packing” of the wear residues as the “third body”, or modification microstructure, or of surface topography; these are considered “polishing” [2].

**Figure 10:**
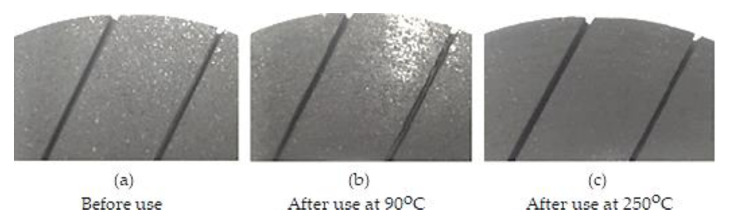
Pads friction material: (**a**) new condition without bedding (0%); (**b**) about 25% bedding; (**c**) approximately 95% bedding.

An example of “bedding”/”polishing” is shown in Figure 10, which shows the friction surface of the brake pad on a disk from the car front in three situations: before use, the intermediate and final period of the polishing/bedding cycle and after an experiment with the test device.

In fact, it is very difficult to capture in a photo the bedding state; in intermediate conditions, (Figure 10b) the bedding zone appears to have a the shiny contact region judging by the light reflection, which would translate as polished. In the final state (95% bedding, Figure 10c), the brake pad friction surface is polished, but it looks rather like a matte surface rather than glossy (making it more difficult to distinguish), but looked at carefully, you can see traces of the rotational movement of the disk as it worked and a layer (brake dust) was bedding on the friction surface of the plate which was the color of graphite, therefore, it was polishing.

A steady-state brake performance is unlikely until the friction surfaces are bedded/polished. However, there are contact effects studies on local heat generation by friction, as well as thermal expansion, friction variation in local contact areas and wear at the brake interface which offer different perspectives on the polishing process [2,23].

The prediction of friction material characteristics of friction and wear by analysis and calculation are not enough; experimental development and testing are essential. Changes in the friction coefficient, *μ* of the disk-pad braking couple must be anticipated and a good design of the system and brake can contribute to the contact effects and minimize these variations. The friction coefficient value, *μ* and any variation associated with the environment and conditions operating, substantially define the braking system, and the level of achievement and performance required of it is a fundamental component of the friction material design and verification.

Thus, tests of friction materials in the laboratory and in traffic showed that the friction coefficien, m changed within the limits ± 10% of the nominal one; i.e., the performance of *μ* must be evaluated within these limits (upper and lower), when a value of *μ* is used for the brake system design.

Although different methods of testing brakes and/or braking systems may be applied under road or laboratory conditions, all of these involve:

- evaluation of the chemical and physical properties of the friction materials, such as chemical composition, hardness, modulus of elasticity, moisture absorption, oil absorption, etc.;
- friction and wear testing of a small sample (specimen) on various test machines;
- large-scale test benches, often called inertia dynamometers;
- rolling test benches;
- testing on the vehicle in real working conditions (operation) or on the test tracks.

These methods are different in purpose and role but are very important for the stability and performance of the braking system, and for road safety.

## 5. Conclusions

The friction materials represent a car vehicle brake’s essential parts, and vehicle designers must understand the causes of properties variation and the characteristics of tribological behavior. The composition and design of the friction material are as important as the composition and the design of the friction pair body material.

Specifications available for friction materials refer to recommended operating conditions, tribology characteristics, mechanical properties and temperature effects indication. The composition of these materials defines their frictional performance, and manufacturers of car vehicles will use the friction materials couple that meets the conditions requested/imposed by them.

The friction coefficient will change, when the braking load increases and thus, the temperature developed increases significantly at the friction interface, and its limits can be only accurately assessed by testing the friction material.

The friction coefficient value, *μ* and any variation associated with the environment and conditions operating, substantially define the braking system, and the level of achievement and performance required of it is a fundamental component of friction material design and verification.

The friction materials pair most widely used for road vehicles continues to be the resin-bonded composite that rubs on a cast-iron disk. In special conditions other friction materials and friction pairs (e.g., ceramic, carbon) are used that are expensive and whose use is limited (e.g., to high-performance cars).

Assessing the tribological properties of friction systems is a rather complex task, as friction is a typically stochastic process, characterized by a series of random influences, so it is almost impossible to predict the performance and reliability of such a theoretical system in an analytical form.

Experimental methods must be widely used for evaluating brake performance, i.e., testing is the best way to experiment, because the test results in the laboratory are hard to correlate with those in traffic, and in accordance with various regulations relating to road safety. These methods are different in their purpose and role but are very important for the performance, stability and reliability of the braking system as well as road safety.

The evaluation of the friction material in relation to its chemical and/or physical characteristics and friction and wear samples is very convenient for checking the structure and composition conformity, but not is enough to determine the nominal performance of a given brake.

Steady-state brake performaces are unlikely to be reached until the friction surfaces are bedded/polished. Therefore, the designers of braking systems must understand the frictional and wear behavior, the friction materials’ physical–chemical properties and the causes of the variation of in and at the disk-brake pad contact surface.

Once in operation, heat and temperature are the first to affect frictional performance. Although a friction pair basically follows Amontons’ friction laws, the temperatures generated at the friction interface increase significantly when braking duty increases.

The test results can be used to define friction patterns, the influence of structural changes, and of other external, factors and are useful for computational analysis in view of insurance stability and reliability of the braking system.

## Figures and Tables

**Table 1:** Friction coefficient values measured at the disk-pads braking assembly for different materials.

Material	Friction Coefficients Were Measured on a Friction Diameter of 10 mm of a Specimen of Material in Steady-State at a Sliding Speed of ~1 m/s, with an Initial Temperature of 80 °C and an Interface Pressure of ~0.7 N/m^2^
Cast iron with graphite flakes/Resin-bonded composite	0.30–0.40
Soft steel	Friction forces were very variable with a very large range which corresponded to vary variable friction coefficients
Stainless steel/Sintered metal	0.40–0.60

**Table 2:** Lubricants and metals percentage in non-organic asbestos materials (reprinted/adapted with permission from ref. [21]. 2022, Elsevier).

Raw Materials	Ceramic Materials (%)	Materials with a Low Percentage of Steel (%)	Materials with a Low Percentage of Metal (%)	Semi-Metallic Materials (%)
Lubricant				
Fine graphite	4–9	2–8	4–10	4–10
Coarse graphite	2–5	0–7	4–8	5–10
Fine graphite				
Fine graphite	2–5	2–5	2–5	2–7
Copper sulfide	2–5	2–5	2–5	2–10
Other sulfides	0–4	0–4	0–4	2–5
Ferrous metals				
Iron fibres	0	10–18	5–10	20–35
Steel powder	0	2–5	1–2	2–7
Non-ferrous metals				
Copper	10–16	6–15	0–6	2–10
Copper alloys	5–10	5–10	0–5	2–10
Zinc/Tin	0–4	0–4	0–2	0–4

**Table 3:** Mechanical properties of C/C-SiC composites (reprinted/adapted with permission from ref. [21]. 2022, Elsevier).

Bending strength [MPa]	⊥	174 ± 22
	‖	134 ± 31
Compression strength [MPa]	⊥	241 ± 45
	‖	188 ± 36

## Data Availability

Data is contained within the article.
